# Synthesis, crystal structure, Hirshfeld surface and DFT analysis of bis­(4-oxo-4-phenyl­but-2-en-2-olato-κ^2^*O*,*O*′)copper(II)

**DOI:** 10.1107/S2056989025011089

**Published:** 2026-01-01

**Authors:** Kyzlarkhan Siddikova, Sardor Murodov, Daminbek Ziyatov, Dilafruz Jabbarova, Jamshid Ashurov, Shakhlo Daminova

**Affiliations:** a Karshi State Technical University, 225 Mustaqillik Avenue, Karshi City, Kashkadarya region, Uzbekistan; b National University of Uzbekistan named after Mirzo Ulugbek University Street 4 Tashkent 100174 Uzbekistan; c Uzbekistan-Japan Innovation Centre of Youth, University Street 2B, Tashkent, 100095, Uzbekistan; d Institute of Bioorganic Chemistry, Academy of Sciences of Uzbekistan, Mirzo Ulugbek Street 83, Tashkent 100125, Uzbekistan; Universidad de Los Andes Mérida, Venezuela

**Keywords:** crystal structure, benzoyl acetone, Hirshfeld surface, DFT calculation

## Abstract

In the title compound, [Cu(C_10_H_9_O_2_)_2_], which crystallizes in space group *P*2_1_/*n*, the central Cu^II^ ion is four-coordinate and closely approaches an ideal square-planar geometry: Cu—O = 1.9173 (18)–1.920 (2) Å, O—Cu—O = 93.34 (7)°, τ_4_ = 0.00, CShM(square-planar) = 0.085. The crystal packing features offset chains along [011] consolidated by π–π inter­actions [*Cg*⋯*Cg* = 3.1293 (1) Å], weak π–metal contacts [*Cg*⋯Cu = 3.390 (2) Å], and C—H⋯O contacts; Hirshfeld surface analysis shows dominant H⋯H contacts (54.8%), followed by H⋯C (18.8%) and O⋯H (11.3%). DFT (UB3LYP, ECP on Cu) yields *E*(HOMO) = −6.19 eV, *E*(LUMO) = −1.83 eV, Δ*E* = 4.36 eV; the HOMO has a significant metal contribution while the LUMO is ligand π* in character, indicating mixed metal-to-ligand charge-transfer (MLCT)/ligand-centered transitions and high electronic stability.

## Abstract 2

Bis(4-phenyl­butan-2-one-κ^2^*O*,*O*′)copper(II) (*P*2_1_/*n*) features an almost ideal square-planar CuO_4_ core and forms offset chains along [011] consolidated by π–π, weak π–metal and C—H⋯O contacts. Hirshfeld analysis shows dominant H⋯H contacts (54.8%); DFT (UB3LYP, ECP on Cu) gives HOMO/LUMO = −6.19/−1.83 eV (Δ*E* = 4.36 eV), consistent with mixed MLCT/ligand-centered transitions and high electronic stability.

## 1. Chemical context

Classical diketones and related ligands have long been the focus of research, and their propensity to yield diverse and intriguing coordination chemistry is well documented (Tojiboyeva *et al.*, 2025 ▸; Siddikova *et al.*, 2024 ▸, 2025 ▸; Kadirova *et al.*, 2009 ▸). Bis(acetyl­acetonato)copper(II) (Peacock, 1971 ▸) and related bis­(β-diketonato) complexes are formed by coordination of a metal ion to the oxygen atoms of two β-diketonate ligands, yielding two six-membered chelate rings. Owing to the strong chelating ability and tunable electronic properties of β-diketones, these ligands readily form complexes with a variety of transition metals – notably Cu^II^ and Zn^II^ (Radzyminska-Lenarcik & Witt, 2020 ▸), Li (Xie *et al.*, 2023 ▸) and *Ln*^III^ (Atanassova, 2022 ▸) – and have therefore been investigated for applications in remediation of contaminated water and soils. Moreover, acetyl­acetone-derived ligands have been employed to synthesize complexes of the *f*-block metals (Vigato *et al.*, 2009 ▸; Nehra *et al.*, 2022 ▸), broadening their use in extraction, sensing, and functional materials.

Beyond environmental applications, both free β-diketone derivatives and their metal complexes show important biol­ogical activities: anti­bacterial (Rehman *et al.*, 2013 ▸), anti­cancer (Talib *et al.*, 2019 ▸), anti­microbial (Viswanathan *et al.*, 2015 ▸) and anti­fungal (Kashar, 2014 ▸). These effects have been reported for different derivatives and substitution patterns. Importantly, several studies demonstrate that the coordination of β-diketone ligands to transition metals frequently enhances biological activities relative to the uncoordinated ligands (Sheikh *et al.*, 2013 ▸; Yiase *et al.*, 2018 ▸). This increase in potency is commonly attributed to changes in lipophilicity and redox behavior, improved membrane permeability and new modes of inter­action with biomolecular targets introduced by metalation. Thus, in an attempt to improve the therapeutic potential of these compounds, this work describes the properties of bis­(4-phenyl­butan-2-one-κ^2^*O*,*O*′)copper(II) (I).
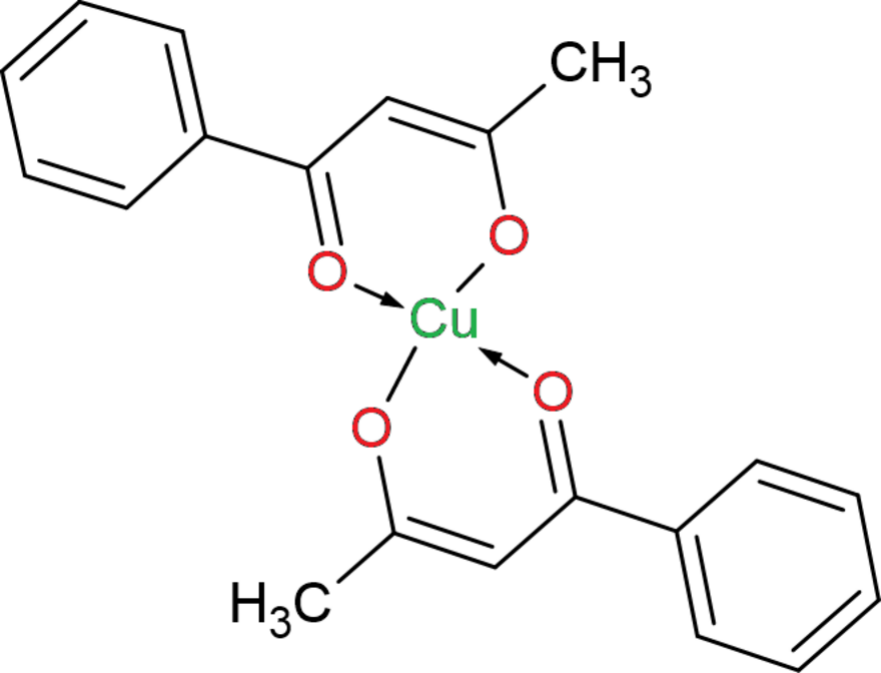


## 2. Structural commentary

**Figure 1:**
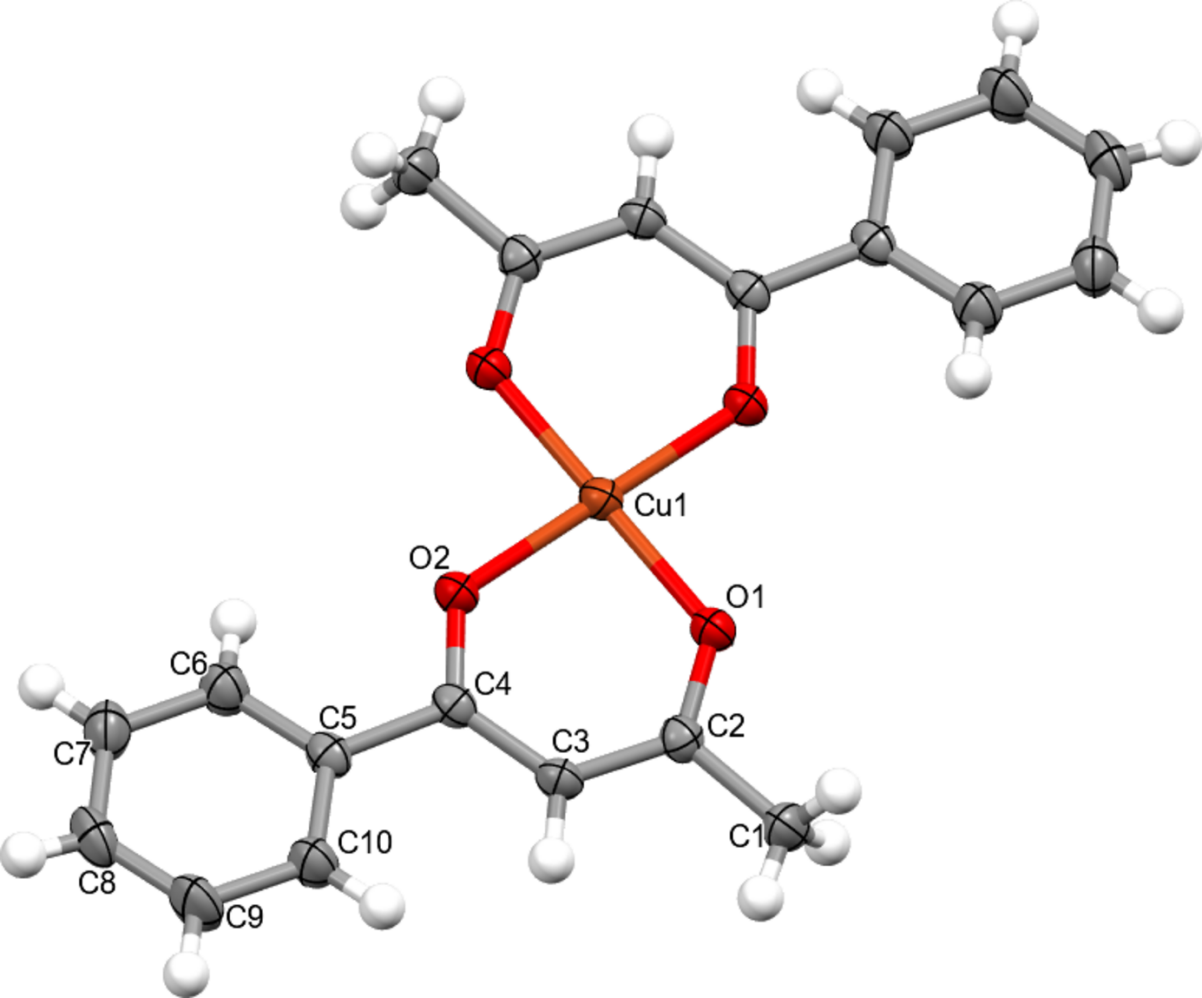
Mol­ecular structure of the title compound with the atom-numbering scheme of the asymmetric unit. Displacement ellipsoids for non-hydrogen atoms are drawn at the 50% probability level. Unlabelled atoms are generated by the symmetry operation −*x* + 2, −*y* + 1, −*z* + 1.

The title compound crystallizes in the monoclinic system, space group *P*2_1_/*n*. The central Cu^II^ atom is four-coordinate and adopts an almost ideal square-planar coordination environment (Fig. 1 ▸). The asymmetric unit contains one mol­ecule of the benzoyl­acetone ligand (BNA), which is coordinated to the copper atom in a bidentate manner through the two carbonyl oxygen atoms.

The bond parameters for the metal–ligand inter­actions are Cu—O1 = 1.9173 (18) Å, Cu—O2 = 1.920 (2) Å, and the chelate angle O1—Cu—O2 = 93.34 (7)° (Table 1 ▸). These values are typical for Cu^II^–β-diketone complexes and indicate an even inter­action of the copper with the two ligand oxygen atoms, without pronounced asymmetry in the bond lengths.

The ligands lie essentially in the same plane; the root-mean-square deviation (RMSD) of the atoms of the ligand fragment from the best plane is 0.122 Å, with the largest deviations observed for atoms C3 = 0.166 (16) Å and C6 = 0.147 (16) Å. For the metal coordination sphere the calculated RMSD = 0.00 Å, reflecting an almost perfect planarity of the O1, O2, O1′, O2′ coordination around Cu^II^.

To qu­anti­tatively assess the degree of distortion of the four-coordinate environment around Cu^II^, the τ_4_ index (Yang *et al.*, 2007 ▸) and the continuous shape measure (CShM) relative to the ideal square-planar geometry (*D*_4*h*_) were calculated. The calculations used four O donors: O1, O2 and their symmetry equivalents (two equivalent bidentate mol­ecules). The results are τ_4_ = 0.00, CShM(square-planar) = 0.085, and CShM(tetra­hedral) = 33.39, which confirms the virtually ideal planarity of the CuO_4_ environment and its proximity to an ideal square-planar geometry.

## 3. Supra­molecular features

**Figure 2:**
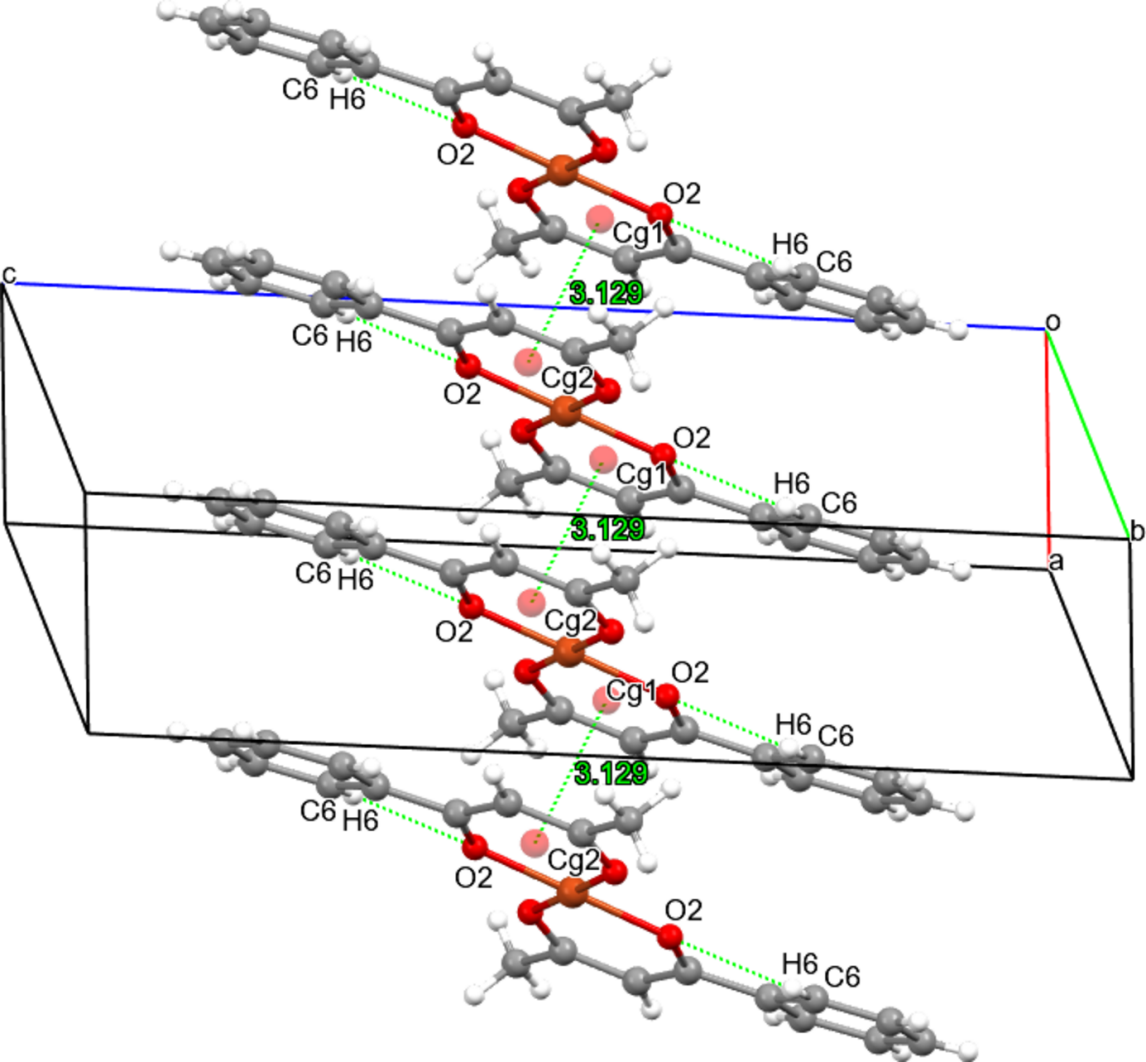
Supra­mol­ecular structure of the title complex showing non-classical C—H⋯O inter­actions and π–π stacking inter­actions, forming chains along [011]. Only hydrogen atoms involved in these inter­actions are shown.

The crystal packing lacks classical strong hydrogen bonds (O—H⋯O, N—H⋯O); however, there are intra­molecular non-standard contacts of the C6—H6⋯O2 type, which can locally stabilize the ligand conformation and slightly influence the geometry of the chelate fragment (Fig. 2 ▸). Such C—H⋯O contacts are often considered weakly directional inter­actions with small bonding energy, but they are important for the local rigidity of the mol­ecule and for precise orientation within the packing (Table 2 ▸).

The mol­ecular packing in the crystal is oriented along the [011] direction and is supported predominantly by π–π stacking between aromatic rings. Two key centroids of aromatic fragments are observed: *Cg*1 (Cu1/O1/C2/C3/C4/O2) and *Cg*2 (Cu1/O1′/C2′/C3′/C4′/O2′), with a centroid–centroid distance *Cg*1⋯*Cg*2 = 3.1293 (1) Å, indicating substantial π-system overlap (Fig. 2 ▸). Additionally, these centroids inter­act with the copper atom: *Cg*1^i^⋯Cu1 = 3.390 (2) Å and *Cg*2^ii^⋯Cu1 = 3.390 (2) Å [symmetry codes: (i) *x* − 1, *y*, *z*; (ii) 1 + *x*, *y*, *z*]. These values demonstrate the presence of weak but structurally significant aromatic ring–metal (π–metal) contacts that contribute to a linear (chain-like) cohesion along the specified direction.

In sum, the packing is governed by a combination of dense H⋯H contacts (a high contribution in the Hirshfeld analysis), H⋯C inter­actions, and directed π–π and π–metal contacts (Fig. 2 ▸). This leads to the formation of offset-oriented chains along [011] with repeating inter­molecular contacts.

## 4. Hirshfeld Surface

**Figure 3:**
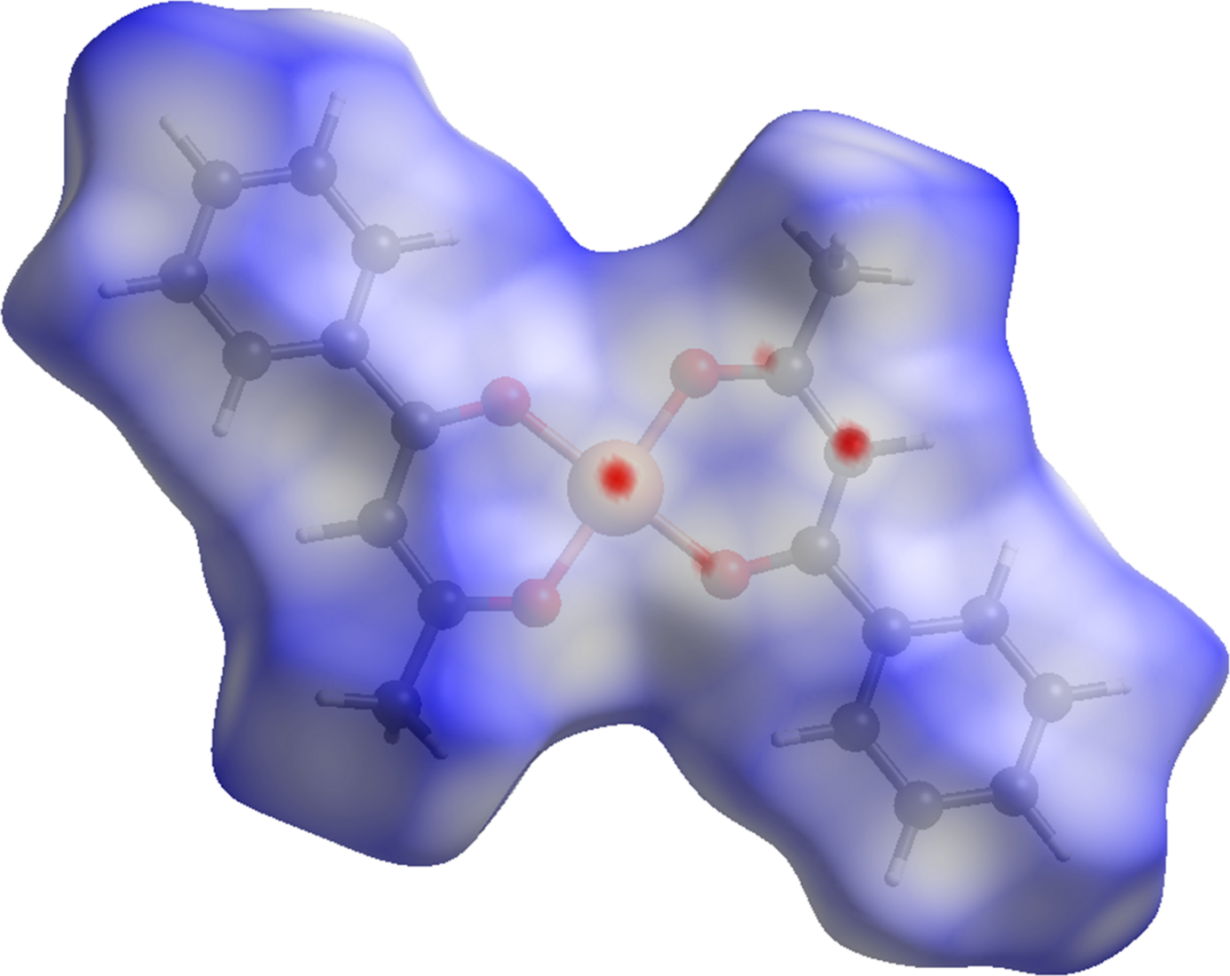
The Hirshfeld surface of the investigated complex, mapped over *d_norm_*. The red spots indicate the shortest inter­molecular contacts in the crystal.

**Figure 4:**
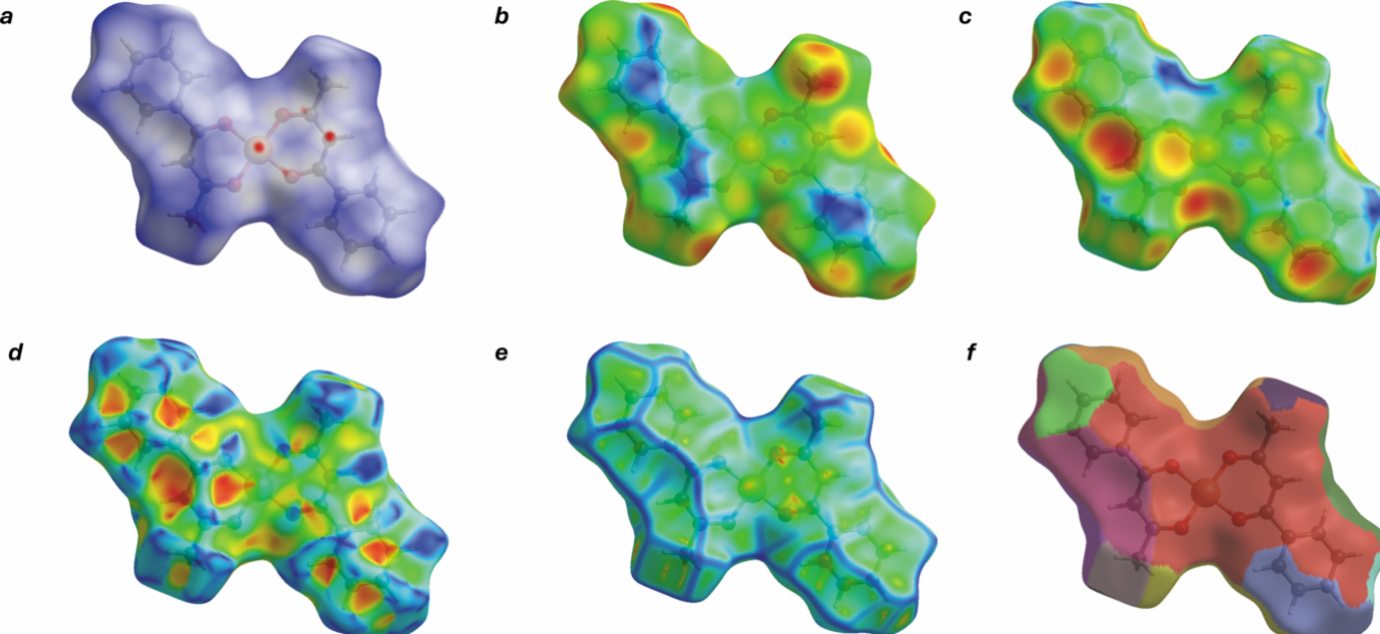
Hirshfeld surfaces of the title complex mapped with (*a*) *d*_norm_, (*b*) *d*_i_, (*c*) *d*_e_, (*d*) shape-index, (*e*) curvedness and (*f*) fragment patch.

The Hirshfeld surface analysis was performed with *CrystalExplorer 21.5* (Spackman *et al.*, 2021 ▸). On the *d*_norm_ map (Fig. 3 ▸), localized red spots indicate contacts shorter than the sum of the van der Waals radii (close contacts), white areas correspond to contacts near the sum of the radii, and blue areas to longer contacts. On the mol­ecular surface in Fig. 4 ▸, flat regions corresponding to the aromatic rings and carbonyl fragments are clearly visible. On the *d*_norm_ map, dark-red spots near O2 and between the rings indicate the closest inter­molecular contacts. The shape index shows paired red–blue regions typical of π–π stacking, with an additional weak signal in the area of the aromatic ring–Cu contact. Curvedness highlights flat areas for the rings and the metal coordination environment and local high-curvature protrusions at the H-substituents. The *d*_e_/*d*_i_ map demonstrates from which side of the surface (inter­nal or external) the inter­actions occur, and the surface breakdown links the localization of the red *d*_norm_ spots to specific mol­ecular fragments (*Cg*1, *Cg*2, O2).

**Figure 5:**
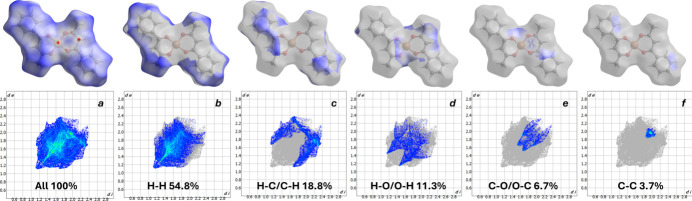
Two-dimensional fingerprint plots for the title compound, showing (*a*) all inter­actions, and decomposed into (*b*) H⋯H, (*c*) H⋯C/C⋯H, (*d*) H⋯O/O⋯H, (*e*) C⋯O/O⋯C and (*f*) C⋯C inter­actions.

The two-dimensional fingerprint plots (Fig. 5 ▸) show that the largest contributions to the total Hirshfeld surface are H⋯H contacts (54.8%) (Fig. 5 ▸*b*), followed by H⋯C/C⋯H (18.8%) (Fig. 5 ▸*c*), H⋯O/O⋯H (11.3%) (Fig. 5 ▸*d*), C⋯O/O⋯C (6.7%) (Fig. 5 ▸*e*) and C⋯C (3.7%) (Fig. 5 ▸*f*). The remaining contributions arise from Cu⋯C/C⋯Cu; Cu⋯H/H⋯Cu and O⋯O, which amount to 2.8%, 0.9% and 1.1%, respectively. These data are consistent with the *d*_norm_ and shape-index maps: the dominance of H⋯H indicates dense mol­ecular packing, the significant H⋯C contribution reflects edge contacts of the aromatic fragments, the O⋯H contribution confirms the presence of intra­molecular and local C—H⋯O contacts, and the presence of distinct red–blue zones on the shape index together with localized red spots on *d*_norm_ in the aromatic-ring regions indicate close π–π and partially π–metal inter­actions.

## 5. DFT calculation

**Figure 6:**
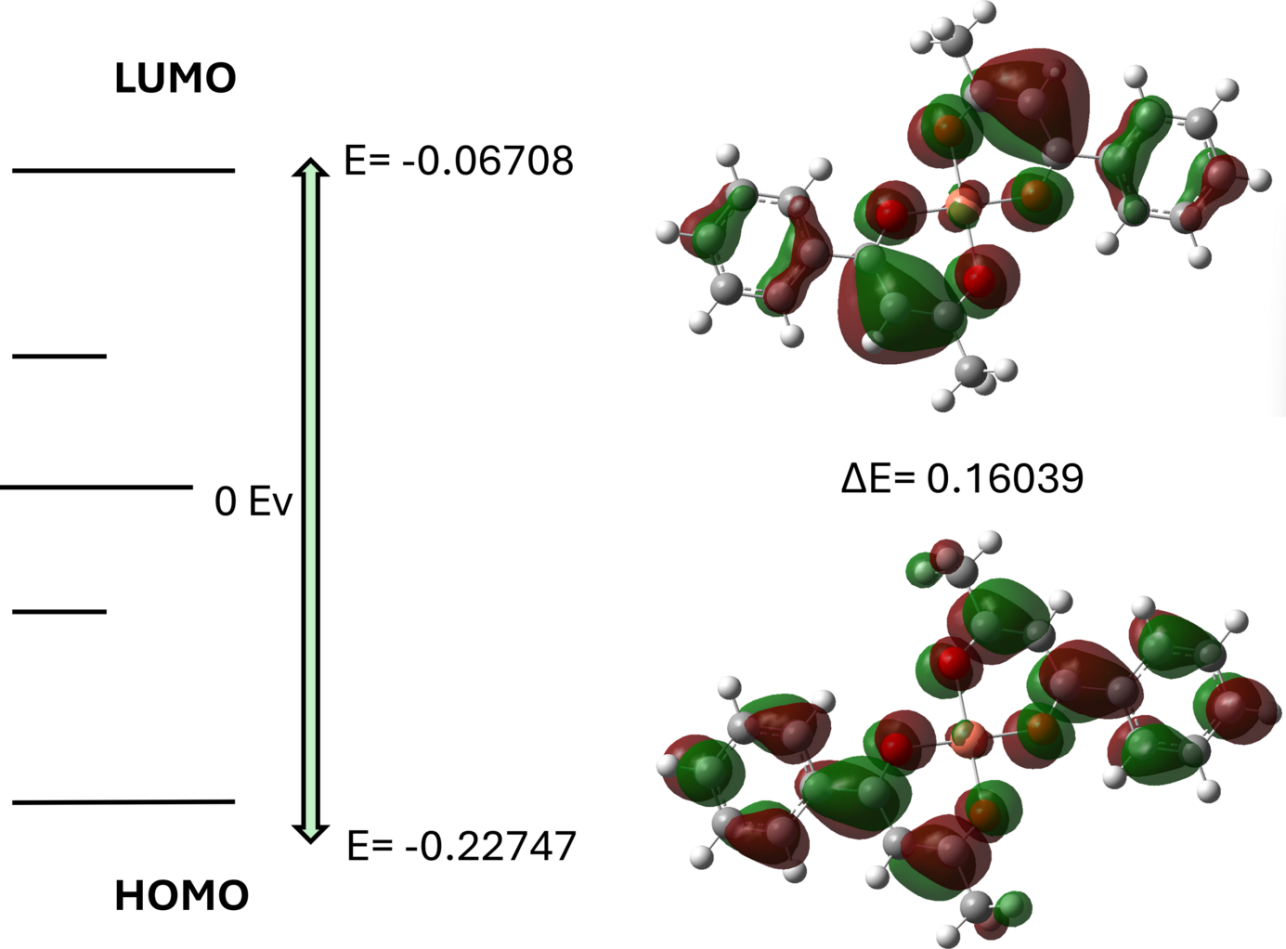
The energy band gap of the title com­pound.

Quantum-chemical calculations were performed with *Gaussian 09* (Revision D.01; Frisch *et al.*, 2016 ▸). Geometry optimizations and frequency calculations were carried out at the UB3LYP level of theory. The Cu atom was described using the LANL2DZ effective core potential and its associated basis set (ECP on Cu = LANL2DZ), while all atoms of the organic fragment (C, H and O) were treated with the 6-311G(d,p) all-electron basis set. Combined basis/ECP definitions were provided via a GenECP section in the Gaussian input. Optimizations were performed without symmetry constraints and frequency analyses confirmed that the optimized geometries correspond to minima (no imaginary frequencies). Calculations were carried out in the gas phase. Charge and multiplicity were set to 0 and 2 (neutral doublet), respectively. The energies of the highest occupied and lowest unoccupied orbitals are *E*(HOMO) = −0.22747 a.u. (−6.19 eV) and *E*(LUMO) = −0.06708 a.u. (−1.83 eV), respectively, yielding an energy gap Δ*E* = *E*(LUMO) - *E*(HOMO) = 0.16039 a.u. (≃ 4.36 eV) (Fig. 6 ▸). Orbital-character analysis shows that the HOMO contains a substantial contribution from the metal center and the nearest donor atoms (metal/metal–ligand bonding character), whereas the LUMO is predominantly localized on the ligand π-system (π*), indicating that the low-energy electronic transitions have metal→ligand charge-transfer (MLCT) character or a mixed ligand-centered/MLCT nature. Conceptual-DFT descriptors derived from the frontier energies are reported in Table 3 ▸. The relatively large HOMO–LUMO gap (≃ 4.36 eV) points to high electronic stability of the mol­ecule and, consequently, moderate chemical reactivity under standard conditions, while the spatial localization of the HOMO and LUMO supports the inter­pretation of the low-energy optical transitions as having a mixed MLCT/ligand-centered character.

## 6. Database survey

A survey of the Cambridge Structural Database (CSD, 2024.2.0; Groom *et al.*, 2016 ▸) identified ten related structures, most of which contain a single BNA mol­ecule. Several entries were found in which two or more BNA mol­ecules are coordinated, for example CSD refcodes: ATONED (Feng *et al.*, 2010 ▸), HIVCAS (Hosseini *et al.*, 2013 ▸), HUDQUU (Patterson *et al.*, 2015 ▸) and LELRUP (Wang *et al.*, 1993 ▸). A closely related study is that of Ekennia *et al.* (2015 ▸), who examined a series of metals (Zn, Cu, Co and Mn), yielding complexes with BNA.

## 7. Synthesis and crystallization

The following solutions were prepared: (*a*) an ethano­lic solution of CuCl_2_·6H_2_O (1.0 mmol) and (*b*) an ethano­lic solution of benzoyl­acetone (BNA) (2.0 mmol). Solution (*a*) was added to solution (*b*) and the mixture was stirred for 12 h at room temperature with magnetic stirring, yielding a yellow crystalline precipitate. The precipitate was filtered, washed several times with ethanol and air-dried. As the obtained material was readily soluble in DMF, it was recrystallized from this solvent to give well-formed blue single crystals suitable for structural and further physico-chemical investigations.
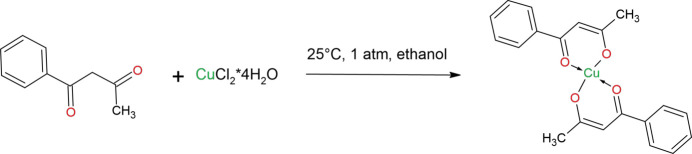


## 8. Refinement

Crystal data, data collection and structure refinement details are summarized in Table 4 ▸. Hydrogen atoms were placed in calculated positions and refined using a riding model with C—H = 0.93–0.98 Å and *U*_iso_(H) = 1.2*U*_eq_(C).

## Supplementary Material

Crystal structure: contains datablock(s) I. DOI: 10.1107/S2056989025011089/dj2086sup1.cif

Structure factors: contains datablock(s) I. DOI: 10.1107/S2056989025011089/dj2086Isup2.hkl

CCDC reference: 2514420

Additional supporting information:  crystallographic information; 3D view; checkCIF report

## Figures and Tables

**Table 1:** Selected geometric parameters (Å, °)

Cu1—O2^i^	1.9173 (18)	Cu1—O1^i^	1.920 (2)
			
O2—Cu1—O1	93.34 (7)		

**Table 2:** Hydrogen-bond geometry (Å, °)

*D*—H⋯*A*	*D*—H	H⋯*A*	*D*⋯*A*	*D*—H⋯*A*
C6—H6⋯O2	0.90 (3)	2.42 (3)	2.736 (4)	101 (2)

**Table 3:** Calculated energies for compound (I)

Total energy, *TE* (eV)	−34 568.6955
*E*(HOMO) (eV)	−6.1898
*E*(LUMO) (eV)	−1.8253
Gap, Δ*E* (eV)	4.3644
Dipole moment, μ(Debye)	0.0000
Ionization potential, *I* (eV)	6.1898
Electron affinity, *A*	1.8253
Electronegativity, χ	4.0076
Hardness, η	2.1822
Electrophilicity index, ω	3.681
Softness, σ	0.4584
Fraction of electron transferred, Δ*N*	−1.836

**Table 4:** Experimental details

Crystal data	
Chemical formula	[Cu(C_10_H_9_O_2_)_2_]
*M*_r_	385.88
Crystal system, space group	Monoclinic, *P*2_1_/*n*
Temperature (K)	293
*a*, *b*, *c* (Å)	4.46365 (18), 10.6100 (5), 18.4333 (7)
β (°)	96.324 (4)
*V* (Å^3^)	867.67 (6)
*Z*	2
Radiation type	Cu *K*α
μ (mm^−1^)	1.96
Crystal size (mm)	0.43 × 0.25 × 0.12

Data collection	
Diffractometer	XtaLAB Synergy, Single source at home/near, HyPix3000
Absorption correction	Multi-scan (*CrysAlis PRO*; Rigaku OD, 2020 ▸)
*T*_min_, *T*_max_	0.916, 1.000
No. of measured, independent and observed [*I* > 2σ(*I*)] reflections	4041, 1660, 1267
*R*_int_	0.040
(sin θ/λ)_max_ (Å^−1^)	0.615

Refinement	
*R*[*F*^2^ > 2σ(*F*^2^)], *wR*(*F*^2^), *S*	0.042, 0.106, 1.03
No. of reflections	1660
No. of parameters	151
H-atom treatment	All H-atom parameters refined
Δρ_max_, Δρ_min_ (e Å^−3^)	0.24, −0.37

Computer programs: *CrysAlis PRO* (Rigaku OD, 2020 ▸), *SHELXT* (Sheldrick, 2015*a* ▸), *SHELXL2018/3* (Sheldrick, 2015*b* ▸) and *OLEX2* (Dolomanov *et al.*, 2009 ▸).

## supplementary crystallographic information

### Bis(4-oxo-4-phenylbut-2-en-2-olato-κ2O,O')copper(II) Crystal data

**Table:**

[Cu(C_10_H_9_O_2_)_2_]	*F*(000) = 398
*M**_r_* = 385.88	*D*_x_ = 1.477 Mg m^−^^3^
Monoclinic, *P*2_1_/*n*	Cu *K*α radiation, λ = 1.54184 Å
*a* = 4.46365 (18) Å	Cell parameters from 1461 reflections
*b* = 10.6100 (5) Å	θ = 4.8–69.9°
*c* = 18.4333 (7) Å	µ = 1.96 mm^−^^1^
β = 96.324 (4)°	*T* = 293 K
*V* = 867.67 (6) Å^3^	Rhombohedral, clear yellowish yellow
*Z* = 2	0.43 × 0.25 × 0.12 mm

### Bis(4-oxo-4-phenylbut-2-en-2-olato-κ2O,O')copper(II) Data collection

**Table:**

XtaLAB Synergy, Single source at home/near, HyPix3000 diffractometer	1660 independent reflections
Radiation source: micro-focus sealed X-ray tube, PhotonJet (Cu) X-ray Source	1267 reflections with *I* > 2σ(*I*)
Mirror monochromator	*R*_int_ = 0.040
Detector resolution: 10.0000 pixels mm^-1^	θ_max_ = 71.4°, θ_min_ = 4.8°
ω scans	*h* = −5→4
Absorption correction: multi-scan (CrysAlisPro; Rigaku OD, 2020)	*k* = −12→12
*T*_min_ = 0.916, *T*_max_ = 1.000	*l* = −21→22
4041 measured reflections	

### Bis(4-oxo-4-phenylbut-2-en-2-olato-κ2O,O')copper(II) Refinement

**Table:**

Refinement on *F*^2^	Primary atom site location: dual
Least-squares matrix: full	Hydrogen site location: difference Fourier map
*R*[*F*^2^ > 2σ(*F*^2^)] = 0.042	All H-atom parameters refined
*wR*(*F*^2^) = 0.106	*w* = 1/[σ^2^(*F*_o_^2^) + (0.0436*P*)^2^ + 0.2379*P*] where *P* = (*F*_o_^2^ + 2*F*_c_^2^)/3
*S* = 1.03	(Δ/σ)_max_ = 0.007
1660 reflections	Δρ_max_ = 0.24 e Å^−^^3^
151 parameters	Δρ_min_ = −0.37 e Å^−^^3^
0 restraints	

### Bis(4-oxo-4-phenylbut-2-en-2-olato-κ2O,O')copper(II) Special details

**Table:**

Geometry. All esds (except the esd in the dihedral angle between two l.s. planes) are estimated using the full covariance matrix. The cell esds are taken into account individually in the estimation of esds in distances, angles and torsion angles; correlations between esds in cell parameters are only used when they are defined by crystal symmetry. An approximate (isotropic) treatment of cell esds is used for estimating esds involving l.s. planes.

### Bis(4-oxo-4-phenylbut-2-en-2-olato-κ2O,O')copper(II) Fractional atomic coordinates and isotropic or equivalent isotropic displacement parameters (Å^2^)

**Table:**

	*x*	*y*	*z*	*U*_iso_*/*U*_eq_	
Cu1	1.000000	0.500000	0.500000	0.0418 (2)	
O2	0.8393 (4)	0.49090 (18)	0.59213 (10)	0.0442 (5)	
O1	0.7796 (4)	0.65046 (19)	0.47100 (10)	0.0466 (5)	
C4	0.6400 (6)	0.5632 (3)	0.61438 (14)	0.0400 (6)	
C2	0.5798 (6)	0.7017 (3)	0.50565 (14)	0.0427 (7)	
C3	0.5024 (7)	0.6610 (3)	0.57305 (15)	0.0445 (7)	
C1	0.4233 (10)	0.8132 (4)	0.4694 (2)	0.0614 (10)	
C8	0.4508 (9)	0.4909 (4)	0.83225 (19)	0.0661 (10)	
C5	0.5642 (6)	0.5375 (3)	0.69003 (15)	0.0425 (6)	
C10	0.4113 (8)	0.6245 (4)	0.72828 (18)	0.0591 (9)	
C7	0.6015 (9)	0.4038 (4)	0.7954 (2)	0.0662 (10)	
C6	0.6607 (8)	0.4277 (4)	0.72467 (18)	0.0580 (8)	
C9	0.3552 (9)	0.6004 (4)	0.7991 (2)	0.0734 (11)	
H8	0.431 (8)	0.473 (3)	0.875 (2)	0.069 (11)*	
H3	0.350 (6)	0.704 (3)	0.5899 (14)	0.045 (8)*	
H1A	0.294 (11)	0.844 (5)	0.495 (3)	0.125 (19)*	
H9	0.258 (9)	0.661 (4)	0.826 (2)	0.092 (13)*	
H6	0.749 (7)	0.369 (3)	0.6988 (18)	0.067 (10)*	
H1B	0.555 (10)	0.862 (5)	0.456 (3)	0.119 (19)*	
H1C	0.306 (15)	0.786 (6)	0.427 (4)	0.19 (3)*	
H7	0.667 (8)	0.332 (4)	0.8167 (19)	0.075 (12)*	
H10	0.350 (8)	0.700 (4)	0.7066 (18)	0.076 (12)*	

### Bis(4-oxo-4-phenylbut-2-en-2-olato-κ2O,O')copper(II) Atomic displacement parameters (Å^2^)

**Table:**

	*U*^11^	*U*^22^	*U*^33^	*U*^12^	*U*^13^	*U*^23^
Cu1	0.0430 (3)	0.0454 (4)	0.0387 (3)	0.0051 (3)	0.0118 (2)	−0.0004 (3)
O2	0.0472 (11)	0.0483 (12)	0.0385 (10)	0.0081 (9)	0.0105 (8)	0.0009 (9)
O1	0.0489 (11)	0.0476 (11)	0.0458 (11)	0.0055 (10)	0.0160 (9)	0.0032 (10)
C4	0.0424 (14)	0.0419 (15)	0.0363 (14)	−0.0031 (12)	0.0069 (11)	−0.0064 (12)
C2	0.0454 (15)	0.0404 (15)	0.0428 (15)	−0.0025 (12)	0.0066 (13)	−0.0005 (13)
C3	0.0480 (15)	0.0460 (16)	0.0414 (15)	0.0072 (13)	0.0128 (12)	−0.0039 (14)
C1	0.069 (2)	0.049 (2)	0.070 (2)	0.0142 (18)	0.026 (2)	0.0156 (19)
C8	0.078 (2)	0.084 (3)	0.0392 (17)	−0.007 (2)	0.0203 (16)	0.004 (2)
C5	0.0436 (14)	0.0480 (16)	0.0365 (14)	−0.0040 (12)	0.0067 (11)	−0.0048 (13)
C10	0.073 (2)	0.058 (2)	0.0492 (18)	0.0118 (18)	0.0221 (16)	0.0006 (17)
C7	0.082 (3)	0.063 (2)	0.055 (2)	0.003 (2)	0.0131 (18)	0.015 (2)
C6	0.073 (2)	0.057 (2)	0.0466 (18)	0.0082 (18)	0.0154 (16)	0.0014 (16)
C9	0.096 (3)	0.078 (3)	0.053 (2)	0.015 (2)	0.036 (2)	−0.002 (2)

### Bis(4-oxo-4-phenylbut-2-en-2-olato-κ2O,O')copper(II) Geometric parameters (Å, º)

**Table:**

Cu1—O2^i^	1.9173 (18)	C1—H1C	0.94 (7)
Cu1—O2	1.9173 (18)	C8—C7	1.367 (5)
Cu1—O1^i^	1.920 (2)	C8—C9	1.359 (5)
Cu1—O1	1.920 (2)	C8—H8	0.82 (4)
O2—C4	1.276 (3)	C5—C10	1.386 (4)
O1—C2	1.275 (3)	C5—C6	1.375 (5)
C4—C3	1.390 (4)	C10—C9	1.380 (5)
C4—C5	1.496 (4)	C10—H10	0.92 (4)
C2—C3	1.394 (4)	C7—C6	1.382 (5)
C2—C1	1.494 (5)	C7—H7	0.89 (4)
C3—H3	0.90 (3)	C6—H6	0.90 (3)
C1—H1A	0.85 (5)	C9—H9	0.94 (4)
C1—H1B	0.84 (5)		
			
Cg1···Cg2^ii^	3.1293 (1)	Cu···Cg1^ii^	3.390 (2)
Cg2···Cg1^iii^	3.1293 (1)	Cu···Cg2^iv^	3.390 (2)
			
O2^i^—Cu1—O2	180.0	H1A—C1—H1C	103 (5)
O2—Cu1—O1	93.34 (7)	H1B—C1—H1C	106 (5)
O2^i^—Cu1—O1^i^	93.34 (7)	C7—C8—H8	116 (3)
O2^i^—Cu1—O1	86.66 (7)	C9—C8—C7	120.2 (3)
O2—Cu1—O1^i^	86.66 (7)	C9—C8—H8	124 (3)
O1—Cu1—O1^i^	180.00 (7)	C10—C5—C4	121.9 (3)
C4—O2—Cu1	126.63 (18)	C6—C5—C4	119.7 (3)
C2—O1—Cu1	125.48 (18)	C6—C5—C10	118.4 (3)
O2—C4—C3	123.7 (2)	C5—C10—H10	120 (2)
O2—C4—C5	115.3 (2)	C9—C10—C5	120.6 (4)
C3—C4—C5	121.0 (2)	C9—C10—H10	120 (2)
O1—C2—C3	124.8 (3)	C8—C7—C6	120.0 (4)
O1—C2—C1	115.6 (3)	C8—C7—H7	121 (2)
C3—C2—C1	119.7 (3)	C6—C7—H7	119 (2)
C4—C3—C2	125.6 (3)	C5—C6—C7	120.8 (3)
C4—C3—H3	119.6 (18)	C5—C6—H6	118 (2)
C2—C3—H3	114.8 (18)	C7—C6—H6	121 (2)
C2—C1—H1A	112 (3)	C8—C9—C10	120.2 (3)
C2—C1—H1B	108 (3)	C8—C9—H9	119 (2)
C2—C1—H1C	109 (4)	C10—C9—H9	121 (2)
H1A—C1—H1B	118 (5)		
			
Cu1—O2—C4—C3	1.2 (4)	C3—C4—C5—C10	−15.1 (4)
Cu1—O2—C4—C5	−177.83 (17)	C3—C4—C5—C6	168.3 (3)
Cu1—O1—C2—C3	4.4 (4)	C1—C2—C3—C4	−176.5 (3)
Cu1—O1—C2—C1	−175.7 (3)	C8—C7—C6—C5	−1.4 (6)
O2—C4—C3—C2	−6.4 (5)	C5—C4—C3—C2	172.5 (3)
O2—C4—C5—C10	163.9 (3)	C5—C10—C9—C8	0.3 (6)
O2—C4—C5—C6	−12.7 (4)	C10—C5—C6—C7	1.2 (5)
O1—C2—C3—C4	3.4 (5)	C7—C8—C9—C10	−0.4 (6)
C4—C5—C10—C9	−177.3 (3)	C6—C5—C10—C9	−0.7 (5)
C4—C5—C6—C7	178.0 (3)	C9—C8—C7—C6	0.9 (6)

Symmetry codes: (i) −*x*+2, −*y*+1, −*z*+1; (ii) *x*−1, *y*, *z*; (iii) −*x*+1, −*y*+1, −*z*+1; (iv) *x*+1, *y*, *z*.

### Bis(4-oxo-4-phenylbut-2-en-2-olato-κ2O,O')copper(II) Hydrogen-bond geometry (Å, º)

**Table:**

*D*—H···*A*	*D*—H	H···*A*	*D*···*A*	*D*—H···*A*
C6—H6···O2	0.90 (3)	2.42 (3)	2.736 (4)	101 (2)
